# Sir Joseph Lister's Address

**Published:** 1896-09-26

**Authors:** 


### The Hospital

An Institutional Journal of

The Medical Sciences and Hospital Administration, with

Vol. XX.—No. 522.] AN EXTRA NURSING SUPPLEMENT. [Sept. 26, 1896.

### Sir Joseph Lister's Address.

The address delivered at Liverpool by Sir Joseph Lister, as President of the British Association for the Advancement of Science, constitutes the most remarkable demonstration of the claims of the medical profession to high scientific rank which has ever yet been placed before the public. In order to appreciate its full value, it must be remembered that Sir Joseph is not only President of the Association, and an eminent practitioner of surgery, but that, as President of the Royal Society, he is also the official head of science in the United Kingdom, and is, therefore, the one person who is officially entitled to speak with authority concerning all its aspects, many and various as they are. He modestly hinted, indeed, that his selection of a subject was mainly determined by his intimate practical knowledge of its details, as well as of the principles to which they must be referred; but all who know him are aware that he could have discoursed on many other subjects with equal ease and perspicuity, and that the interdependence of science and the healing art would not have been chosen, unless its magnitude and importance had been such as entirely to justify the choice. It is a fitting subject of national pride, more especially at a time when the foreign charlatan is much in the ascendant among fashionable people, to remember the pre-eminent position, in the advancement of medicine and surgery, which has been held, certainly for the last two hundred years, by the subjects of the United Kingdom. Mr. Brudenell Carter, writing in 1857,* used the following words: "The part played by England and her people in the advancement of medical science has been greater, not only than that of any other country, but than that of all other countries put together. In the way of preventive medicine we stand absolutely alone; a few continental researches being just of sufficient value and importance to illustrate the height of our pre-eminence. In the way of curative medicine, the modern temple of Æsculapius may be said to rest upon three discoveries which have constituted epochs—upon the discovery of the circulation, the discovery of the functions of the spinal cord, and the discovery of reflex action—and all of these are English. The application of cleanliness to surgery is English. In all countries there have been felicitous applications of knowledge, suggestions which have lightened the difficulties of surgery, and between which it would be impossible to institute comparisons; but it may be questioned whether the whole of them are not outweighed, in value to the human race, in the relief of suffering and the preservation of life, by the one English achievement of the successful removal of ovarian tumours." When these words were written, it would appear from other parts of the essay that the writer entertained some doubt as to the precise nature of the noxious agent which it was the object of cleanliness in surgery to exclude, whether this might be a living germ or a chemical product; but the progress of bacteriology has long since removed all possibility of uncertainty upon the point.

Sir Joseph describes, with the force and clearness which are derived from simplicity, the gradual growth in his mind of a conviction that the inflammations which so often frustrated the endeavours of the surgeon to obtain the primary union of wounds was due to decomposition or putrefaction of blood within the wound, as well as of the hopelessness of any endeavours to prevent such putrefaction so long as he accepted the received teaching of Liebig, who believed the immediate cause of putrefaction to be the oxygen of the atmosphere, an agent which no chemicals could neutralise, and which no dressings could exclude. It was only when Pasteur had shown that putrefaction was a fermentation caused by the growth of microbes, and that these could not arise *de novo* in the decomposable substance, but must be introduced from without, that the problem assumed a more hopeful aspect. The address explains the selection of carbolic acid as a material by which it was hoped that microbes might be destroyed without injury to the living tissues, and describes the "joy" with which, under its influence, Sir Joseph saw compound fractures follow the same "safe and tranquil course" as simple ones, as well as the new interest arising from the power of observing the course and progress of reparative changes of a kind which had previously always been concealed from view. The application of the new method of dressing to wounds designedly created followed as a matter of course; and then years were spent, years of constant improvement in matters of detail, in separating the essential from the non-essential portions of the original procedure. As a result of this work, Sir Joseph describes how, at the Berlin Congress of 1890, he was able to furnish absolute demonstration of the harmlessness of atmospheric dust in surgical operations, so that from thenceforward irritation of the wound by antiseptic irrigation and washing were avoided, and nature was left undisturbed to carry out her best methods of repair, while the surgeon conducted his operations as simply as in former days, provided always that, deeply impressed with the tremendous importance of his object, and imparting the same conviction to his assistants, he vigilantly maintained from first to last, with a care that once learnt becomes instinctive, but for the want of which nothing else can compensate, the use of the simple means which will suffice to exclude from the wound the coarser forms of septic impurity. From this point the address dealt chiefly with the daily growing importance of bacteriology in medicine, with the researches which had led to the identification of the bacilli of diphtheria, of cholera, and of tubercle, with the separation of the "toxins" formed by the several bacilli, and with the nature and uses of the "anti-toxins," which are becoming daily of more and more importance in the prevention and treatment of bacterial diseases. It is worthy of note that Sir Joseph appears to be fully convinced of the efficacy of the anti-toxins, although the point is one on which it cannot be said that entire agreement has been arrived at among physicians.

We are not acquainted with any statistics which are at once sufficiently extensive and sufficiently precise to give any adequate conception of what has been the value, to the human race, of the work done by Sir Joseph Lister and by those who have followed in his footsteps. The address itself makes mention of the disappearance of hospital gangrene and related maladies from the great General Hospital of Vienna, where formerly 80 per cent. of the wounds became affected by them; and the *Times*, in commenting upon it, said that, as a result of the adoption of "Listerism," surgery could now, "almost without risk, remove any part of the body, external or internal, which is not of itself essential to the continuance of life." A very considerable number of internal or visceral diseases of frequent occurrence, such as renal calculus, impacted gallstones, intestinal obstruction, and many others, were, only a few years ago, practically incurable; and, although they might sometimes terminate in recovery, more frequently led through illness, often prolonged and always painful, to a death which was welcomed as a relief from suffering. They were known to be due, for the most part, to simple mechanical conditions which the natural forces were unable to overcome, but which would have been quite easy of removal by operation if it had not been that openings of the great cavities of the body were almost invariably rendered fatal by the occurrence of spreading septic inflammation. All such conditions are remedied now almost as a matter of course; and a surgeon who allowed his patient to die unrelieved from any of them would be justly regarded as having failed in his first duty. When we consider that, as a direct result of Sir Joseph Lister's work, the number of surgical operations, and the number of conditions remediable by operation, have immensely increased, that danger to life from the actual operation has almost disappeared, and that these consequences, instead of being confined to Great Britain, have been extended over the whole world, it becomes apparent that the lives which have been rescued from misery or saved from death must already be counted by thousands, and that the tale is daily increasing. The discovery of the nature of fermentation was, indeed, wholly due to Pasteur; but it was reserved for Lister to apply Pasteur's discovery in a new region, and for the attainment of ends the possibility of which only a surgeon could have foreseen. And, while we give full credit to Lister, we must not forget that the extension of his work has been effected by an army of surgical fellow-labourers, and that it is not only to him, but to the whole surgical profession working under his guidance, that the relief of pain and the prolongation of life are to be attributed. Thirty-one years have passed away since Lister first applied a carbolised dressing to a compound fracture; and, in the intervening time, the world has witnessed a revolution in surgery, compared to which the revolution in commerce effected by steam navigation, by railroads, and by telegraphs, must be regarded as comparatively insignificant. If there be anything more remarkable than the change itself, it is the indifference with which it is regarded by the public. In any other department of human activity a man who preserved thousands of lives would be regarded as an universal benefactor, for whom no rewards or honours could be excessive, or even adequate. This great work has been done by a surgeon and by his followers, and the world has given but scant appreciation and still scantier recompense as its sole recognition of their labours. It may possibly be regarded as a tribute to the medical profession that its best should always be expected from it, and that its best should be expected to be so good; but in an age which witnesses the conversion of successful tradesmen into hereditary legislators, the facts look more as if they were the outcome of an ingratitude which, in its turn, is in large measure the outcome of ignorance and stupidity.

